# Predictive value of MEP1A in cancer prognosis: A protocol for systematic review and meta-analysis

**DOI:** 10.1097/MD.0000000000023120

**Published:** 2020-11-06

**Authors:** Yong Chen, Fangfang Wu, Li Zhang, Li Du, Xiang Yan

**Affiliations:** a The First Hospital of Lanzhou University; b Evidence-Based Nursing Center, School of Nursing, Lanzhou University; c The Third Ward of Cardiovascular Clinical Medical Center, Affiliated Hospital of Gansu University of Chinese Medicine; d The Third People's Hospital of Lanzhou City, Lanzhou, China.

**Keywords:** cancer, MEP1A, prognosis, systematic review

## Abstract

**Background:**

Meprin is a member of the astaxanthin family; it performs many functions through a wide range of proteolytic enzyme activities during health and disease, including tumors and inflammatory conditions. The purpose of this systematic review was to evaluate the predictive value of MEP1A in tumor prognosis.

**Methods:**

A comprehensive search was conducted on PubMed, Cochrane library, and Web of Science Database using a developed search strategy. The Newcastle-Ottawa Scale (NOS) or the Cochrane Collaboration's tool for assessing risk of bias will be used to access the methodological quality of included studies, and GRADE will be applied to evaluate evidence quality of outcomes. All analyses were performed by Stata 15.0.

**Results:**

The results will systematically summarize and display the currently collected evidence on the predictive value of MEP1A in different tumor prognosis.

**Conclusion:**

This study may play a certain role in predicting the prognosis of cancer patients in the future, and may prompt clinicians to make necessary treatment or prevention plans as soon as possible.

**Ethics and communication:**

It is not necessary because the present systematic review is based on published studies.

**INPLASY registration number:**

INPLASY2020100005.

## 1 Introduction

Meprin is a member of the astaxanthin family. It is a zinc-containing metalloendopeptidase that was discovered as a protease highly expressed on renal brush border cell membrane and intestinal epithelial cells for the first time.^[1,2]^ Meprin performs many functions through a wide range of proteolytic enzyme activities during health and disease, including tumors and inflammatory conditions.^[3–5]^ Meprin is composed of two homologous subunits, including two domains of Meprin-α (MEP1A) and Meprin-β (MEP1B), with 42% amino acid sequence identity between the two.^[6]^ Meprin-α and Meprin-β can cleave many proteins of extracellular matrix, including pro-collagen I, pro-collagen III, fibronectin, and collagen IV.^[7–9]^ MEP1A is expressed differently in different cancer types, such as colorectal cancer (CRC), breast cancer, osteosarcoma, pancreatic cancer, etc.^[10,11]^ There are reports showing that MEP1A has abnormal secretion to the stroma in CRC.^[3]^ In addition, the expression of MEP1A also exists in the human prostate cancer cell line model and helps to promote cell replication and invasion.^[12]^ However, the exact substrate and pathogenic mechanism of Meprin protein in tumors are still unclear.

In order to explore the role of MEP1A in the occurrence and development of diseases, many researchers have studied the potential role of MEP1A in various diseases. Studies have shown that in a cohort of ulcerative colitis (UC) patients, there is a genetic association between MEP1A and inflammatory bowel disease, and the MEP1A knockout mice showed more serious intestinal damage and inflammation than wild-type mice, suggesting that the decreased expression of MEP1A is related to intestinal inflammation.^[13]^ Meanwhile, several studies also have proposed that MEP1A plays an important role in the development of tumors and can be used as a new predictor of tumor prognosis. OuYang et al^[14]^ reported the role and diagnostic value of MEP1A in human hepatocellular carcinoma (HCC). The results of clinical studies showed that compared with paired adjacent nonneoplastic tissues and nontumor liver tissues, the expression level of MEP1A mRNA in HCC was significant increase in tumor tissue. The immunohistochemical analysis was conducted on tissue samples of 394 patients who were with HCC, and the results showed that the positive expression of MEP1A in tumor cells was an important risk factor affecting survival after radical resection. Furthermore, subgroup analysis showed that patients with positive MEP1A expression in tumor cells had a worse surgical prognosis compared with patients with negative MEP1A expression in tumor cells. Wang et al^[6]^ studied the expression and clinicopathological characteristics of MEP1A in CRC, and the outcomes indicated that MEP1A expression was enhanced in CRC, the inhibition of MEP1A expression can inhibit cell proliferation and invasion of CRC in vitro and in vivo. Also, the tumor size, American Joint Committee on Cancer (AJCC) staging, and T and N staging are related to the expression of MEP1A, and MEP1A is an independent prognostic factor of CRC overall survival.^[6]^ MEP1A also has its unique advantages in the diagnosis or differential diagnosis of some tumors. A study^[15]^ focused on the expression of two intestinal markers, galectin-4 and MEP1A in ovarian and gastrointestinal mucinous carcinomas. The study revealed that while galectin-4 expression was relatively consistent in different tumor tissues, the expression of membranous MEP1A in mucinous ovarian carcinomas (MOCs) was significantly lower than that in gastrointestinal cancer. It suggested that MEP1A could be helpful to differentiate primary and secondary ovarian mucinous adenocarcinoma.

In this study, we will search the English database to collect evidence about the application of MEP1A in tumor prognosis, and plan to conduct a systematic review and meta-analysis based on the extracted data, hoping to provide a reference for the prediction of MEP1A in the field of tumor prognosis.

## 2 Methods

### 2.1 Design and registration

A systematic review and meta-analysis for predictive value of MEP1A in cancer prognosis is planned to be completed. Our research protocol has been registered on International Platform of Registered Systematic Review and Meta-analysis Protocols,^[16]^ the register number is INPLASY2020100005, available from: https://inplasy.com/inplasy-2020–10-0005/. The protocol and the full text will be completed according to the items mentioned in Preferred Reporting Items for Systematic Review and Meta-Analysis protocols (PRISMA-P) and PRISMA statements, respectively.

### 2.2 Inclusion criteria

#### 2.2.1 Participants

Patients with common tumors who have been diagnosed by pathology, mainly including gastric cancer, lung cancer, CRC, esophageal cancer, liver cancer, breast cancer, prostate cancer, and some other tumors with a higher incidence or mortality. There are no limitations in age, race, or nationality.

#### 2.2.2 Intervention

Patients with positive/high expression of MEP1A were considered as intervention group.

#### 2.2.3 Comparator

Patients with negative/low expression of MEP1A were considered as control group.

#### 2.2.4 Outcome measures

##### 2.2.4.1 Main outcomes

Overall survival (OS), progression-free survival (PFS), disease-free survival (DFS), recurrence-free survival (RFS), and disease-specific survival (DSS).

##### 2.2.4.2 Secondary outcomes

Correlations between MEP1A expression and clinicopathological features, such as tumor size, stage, and metastasis.

#### 2.2.5 Study design

Randomized controlled trials, cohort studies, case-control studies, or cross-sectional studies.

### 2.3 Excluded criteria

The following studies will be excluded: duplicate publications, review, comments, case reports, nonhuman study, uncontrolled study, or studies were not in English/Chinese. The data needed to be extracted in the study is incomplete.

### 2.4 Information sources

A comprehensive search was conducted on PubMed, Cochrane library, and Web of Science Database using the combination of medical subject headings (MeSH) and free words. The retrieval time is up to September 21, 2020. The publication language is limited to Chinese and English without time restriction. The search terms mainly related to “MEP1A” and “cancer”. For example, the specific retrieval strategy in PubMed is as follows: (“meprin A”[Supplementary Concept] OR “MEP1A”[Title/Abstract] OR “meprin-α”[Title/Abstract] OR “meprin alpha”[Title/Abstract] OR “Meprin A”[Title/Abstract] OR “Meprin A Subunit Alpha”[Title/Abstract] OR “meprin 1 alpha”[Title/Abstract] OR “Endopeptidase-2”[Title/Abstract] OR “N-benzoyl-L-tyrosyl-P-amino-benzoic acid hydrolase subunit alpha”[Title/Abstract] OR “PABA peptide hydrolase”[Title/Abstract] OR “PPH alpha”[Title/Abstract] OR “PPH α”[Title/Abstract] OR “PPHA”[Title/Abstract]) AND (“neoplasms”[MeSH Terms] OR “neoplasm∗”[Title/Abstract] OR “cancer∗”[Title/Abstract] OR “tumour∗”[Title/Abstract] OR “tumor∗”[Title/Abstract] OR “carcinoma∗”[Title/Abstract]).

### 2.5 Selection of studies

All the retrieved records were imported into EndNote X8, and then the duplicates were removed. First, two independent researchers browsed the titles and abstracts to identify the initially included studies and exclude the apparently unrelated ones. For the study that cannot be determined whether it fully meets the inclusion criteria will be evaluated after reading the full text. The results of literature screening were cross-checked by two identical researchers and judged by a third experienced person if there were disagreements.

### 2.6 Data extraction and management

The spreadsheet for extracting data will be prepared in advance using Microsoft Excel 2016 (Microsoft Corp, Redmond, WA, www.microsoft.com). The data extraction will be performed by two researchers independently and cross-check will be carried out to ensure the consistency and accuracy of the data after completing the previous work. The disagreements in the process should be settled by two persons through discussion or by a third party. The information extracted from eligibility studies mainly includes title, first author, country of corresponding author, year of publication, journal, study design, funding, characteristics of included population, such as sample size, age, gender, disease, follow-up period, tumor size, stage, and metastasis, outcomes such as overall survival (OS), progression-free survival (PFS), disease-free survival (DFS), recurrence-free survival (RFS), disease-specific survival (DSS), hazard ratio (HR)/odds ratio (OR), and their 95% confidence interval (CI). All data analysis will be performed by Stata 15.0 software (StataCorp, College Station, TX, https://www.stata.com).

### 2.7 Evidence quality of outcome measures

Grading of Recommendations Assessment, Development, and Evaluation (GRADE) will be used to access the quality of evidence for each outcome.^[17]^

### 2.8 Quality assessment

The quality assessment of included studies will be appraised using the Newcastle-Ottawa Scale (NOS) for the nonrandomized controlled trials,^[18]^ which includes 8 items in 3 aspects, with a total of 9 points. In this study, studies with scores higher than the median are of high quality (low risk of bias) and vice versa, low quality (high risk of bias). And the Cochrane Collaboration's tool for assessing risk of bias for the randomized controlled trials,^[19]^ which contains 7 items in 6 domains, the specific items are as follows: selection bias (generation of random sequences and allocation concealment), implementation bias (blinding of implementers and participants), measurement bias (blind method in outcome evaluation), loss to follow-up bias (incomplete outcome data), reporting bias (selective reporting of research results), and other sources of bias. The risk of bias for each item can be rated as “high,” “low,” and “unclear”. The above work would be carried out by two persons separately and the results checked by the same two persons. If there are some differences, they will be solved through discussion.

### 2.9 Statistical analysis

The outcome measures will be calculated using hazard ratio (HR) or odds ratio (OR) for extracted dichotomous variables and mean difference (MD) for the continuous variables. When *P* < 0.05 (two-sided), the difference is statistically significant. Pooled estimates of survival outcomes and their 95% CIs will be presented in forest plots by fixed or random effect model. *I*^2^ statistic is used to evaluate the heterogeneity between studies. When the *I*^2^ is less than 50%, it means that the statistical heterogeneity between studies is small, otherwise, it indicates that there is significant statistical heterogeneity which should be addressed or explained by further analyses. If the heterogeneity between the two groups is too large or the number of included studies is limited and quantitative analysis cannot be performed, a descriptive analysis is planned to be conducted.

### 2.10 Assessment of publication bias

When the number of included studies is greater than 9, Egger's test will be utilized to detect publication bias.

### 2.11 Sensitivity analysis

The study which has a very low-quality may lead to a high risk of bias, so sensitivity analysis will be performed by excluding the low-quality study from further analysis to test if they will influence the stability of the results.

### 2.12 Subgroup analysis

If necessary, subgroup analysis will be conducted based on tumor type, race, and age of included population, and other factors that may affect the robustness of meta-analysis results.

## 3 Results

**Figure 1:**
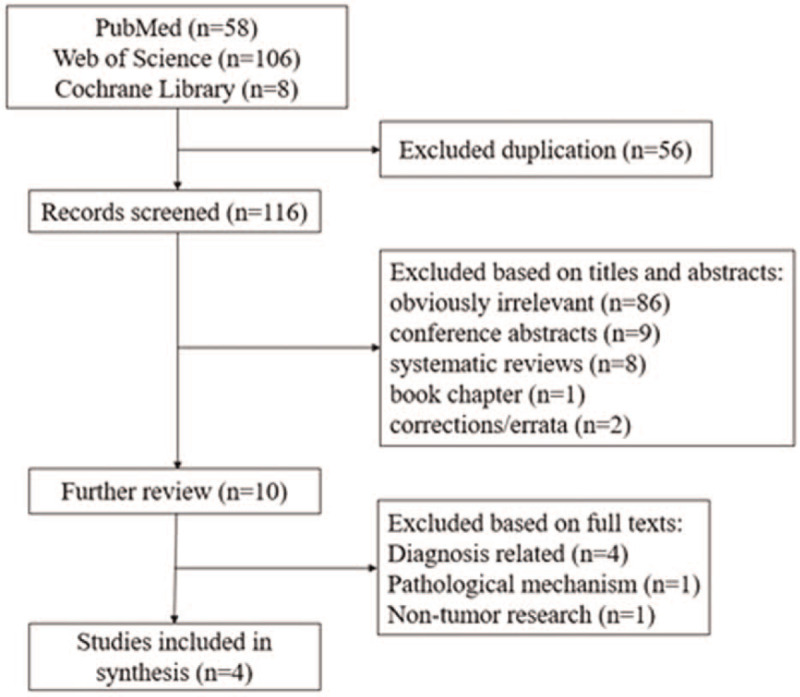
Flow chart of literature screening.

Based on the designed retrieval strategy, a total of 172 records were obtained from PubMed (n = 58), Web of Science (n = 106), and Cochrane Library (n = 8) databases. First, 56 duplicate documents were removed. By reviewing the titles and abstracts of the remaining publications, 87 obviously irrelevant ones were excluded. In addition, 9 conference abstracts, 8 systematic reviews, 1 book chapter, and 2 corrections/errata were also excluded. The remaining 10 studies that are uncertain whether they should be included have been further screened through full-text reading. Finally, a total of 4 studies were identified (Fig. 1).

## 4 Discussion

At present, some studies have reported that MEP1A has been involved in the diagnosis and prognosis prediction of different kinds of diseases, including tumor and nontumor. But in fact, on the whole, there are not many such studies. For its role in nontumor diseases, for instance, Chen et al concluded that when serum MEP1A is greater than 12.95 ng/ml, the sensitivity and specificity of predicting intravenous immunoglobulin ineffective Kawasaki disease (KD) reached 80%, indicating that it may take part in the pathogenesis of KD, and can be used as an index to evaluate the disease activity of KD, and can better predict immunoglobulin ineffective KD.^[20]^ In addition, another study which aimed to explore the relationship between the plasma levels of brain natriuretic peptide (BNP) and MEP1A protease and the severity of coronary artery disease found that the levels of BNP and MEP1A in peripheral blood of patients with coronary heart disease were increased, and the higher the levels of BNP and MEP1A, the higher the risk of acute coronary syndrome (ACS).^[21]^ Through these research evidences, MEP1A expression level is more and more promising as a potential marker for the diagnosis of multiple diseases. Similarly, in the field of cancer, the application of MEP1A is not limited to a specific tumor and it has been reported in several studies that MEP1A is highly expressed in various tumors, when it is combined with the different clinical and pathological characteristics of each tumor, it shows an essential value for diagnosis and prognosis. The clinicopathological characteristics of tumors such as gender, age, tumor size, metastasis, TNM (tumor node metastasis) staging, etc are all important clinical and biological characteristics of tumors. Therefore, although the studies of OuYang et al^[14]^ and Wang et al^[6]^ have shown that high expression of MEP1A is a relatively common phenomenon in different tumors, due to the clinicopathological characteristics are different for different types of tumors, which can help to further observe the development of tumors and their own different characteristics.

## Acknowledgment

We are very grateful to Dr Ming Liu for his valuable suggestions on our research methods.

## Author contributions

**Data curation:** Yong Chen, Li Zhang, Li Du.

**Formal analysis:** Yong Chen, Fangfang Wu, Li Zhang.

**Project administration:** Xiang Yan.

**Supervision:** Xiang Yan.

**Writing – original draft:** Yong Chen, Fangfang Wu.
